# Comparison of Endoscopic, Radiological, and High-Resolution Manometry Findings with Endosonographic Findings in Patients with Dysphagia

**DOI:** 10.5152/tjg.2025.24573

**Published:** 2025-07-16

**Authors:** Aslıhan Bayır, Elif Yorulmaz, Deniz Öğütmen Koç, Hatice Yorulmaz

**Affiliations:** 1 Department of Internal Medicine, University of Health Sciences, Bağcılar Training and Research Hospital, Istanbul, Türkiye; 2 Clinic of Internal Medicine, Bakirkoy Dr. Sadi Konuk Training and Research Hospital, Istanbul, Türkiye; 3 Department of Gastroenterology, University of Health Sciences, Bağcılar Training and Research Hospital, Istanbul, Türkiye; 4 Department of Gastroenterology, University of Health Sciences, Gaziosmanpaşa Training and Research Hospital, Istanbul, Türkiye; 5 Department of Physiology, Haliç University Faculty of Medicine, İstanbul, Türkiye

**Keywords:** Achalasia, dysphagia, endosonography, high resolution manometry

## Abstract

**Background/Aims:**

The aim of this study is to reveal the contribution and clinical usability of endosonography in the differential diagnosis of patients presenting with dysphagia.

**Materials and Methods:**

The study included 77 patients with dysphagia. Age, gender, comorbidity, body mass index, history of radiotherapy and chemotherapy, history of trauma, and history of previous head and neck surgery were recorded from the patients’ files. Duration of dysphagia complaints and Eckardt scores were calculated. High-resolution manometry (HRM) and endosonographic ultrasonography (EUS) results were evaluated retrospectively.

**Results:**

Of the patients included in the study, 41 were male and 36 were female. Endosonographic ultrasonography muscularis mucosa, EUS muscularis propria, and EUS total wall thickness values of achalasia patients were found to be higher than those of the healthy control group (*P* < .05). Eckardt scoring of achalasia patients with IRP (Integrated relaxation pressure) pressure below 20 mmHg was higher than those with integrated relaxation pressure (IRP) pressure above 20 mmHg (*P* < .05).

**Conclusion:**

In the differential diagnosis of dysphagia, EUS contributed to the diagnosis of HRM in the detection of malignancy. In addition, EUS esophageal wall thicknesses of achalasia patients were found to be significantly higher than those of the healthy group. Manometric examination of patients with increased esophageal wall thickness in patients undergoing EUS in the differential diagnosis of upper gastrointestinal (GI) pathologies will be useful in terms of motility disorder.

**Main Points**

- Achalasia is basically diagnosed with high-resolution manometry (HRM). Some patients diagnosed with achalasia in HRM may have pseudoachalasia and may have a serious underlying problem such as malignancy.
- Endosonographic ultrasonography (EUS) is a reliable diagnostic tool in the differential diagnosis of cardia tumor and achalasia.
- This studyshowed that EUS provided an additional contribution to HRM diagnosis in the detection of malignancy in the differential diagnosis of dysphagia.

## Introduction

Dysphagia is a symptom that occurs as a result of mechanical obstruction of the passage of food from the mouth to the stomach, decreased strength of the muscles that provide swallowing movement, or impaired coordination due to neuromuscular causes and is an important health problem that severely affects the quality of life of patients. In epidemiologic studies, the prevalence of dysphagia is between 16% and 22% over the age of 50 years.^1^^,2^ There are 2 types of dysphagia: oropharyngeal and esophageal. Major symptoms of oropharyngeal and esophageal dysphagia include difficulty swallowing, odynophagia, regurgitation, pyrosis, and non-cardiac chest pain. Oropharyngeal dysphagia is seen as a result of neuromuscular diseases affecting esophageal upper sphincter function and diseases associated with structural causes related to the oropharynx and larynx. Esophageal dysphagia is caused by major esophageal motility disorders, lymphadenomegaly compressing the esophagus, vascular pathologies, spinal osteophytes, malignancies, esophageal diverticula and rings, dysmotility disorders associated with gastroesophageal reflux disease, foreign bodies, and peptic strictures. Head trauma, cerebrovascular history, Parkinson’s disease, and esophageal malignancy are other conditions in which dysphagia symptoms are common.^1^

Esophageal gastric duodenal graphy (EGD), manometry, and upper gastrointestinal tract (GIS) endoscopy are the first investigations to be performed in the investigation of the etiopathogenesis of dysphagia. It is known that manometry examination is the gold standard for the diagnosis of esophageal motility disorder.^3^ The literature reports that manometry cannot differentiate achalasia from pseudoacalasia and barium esophagogram has limited value in differentiating the 2. Tumors or strictures of the esophagus and cardia can be diagnosed by upper GI endoscopy, but the tumoral structure must invade the submucosal layer to be seen endoscopically. Standard methods are insufficient to diagnose achalasia and malignancy-related pseudoacalasia. Although additional radiologic imaging and endosonography for the diagnosis of pseudoacalacia are of high costs, it is known that there are 4%-5% of pseudoacalacia patients who are diagnosed with achalasia in manometric examination, and the malignancy was not diagnosed during the endoscopic examination.^4^^,^^5^ Therefore, endosonographic ultrasonography (EUS) examination should be performed especially in patients with obstructive patterns on manometry examination and in the presence of high clinical suspicion in favor of malignancy. Esophageal wall thicknesses of patients with achalasia and Jackhammer esophagus, which are among the esophageal motility disorders, were found to be increased when compared to healthy individuals. At the same time, many patients with esophageal motility disorders that are not well defined in manometry have been found to have increased esophageal muscle thickness in EUS examination.^6^^,^^7^ The aim of this study was to investigate whether the pathologies detected in upper GI endoscopy, EGD, and high-resolution manometry (HRM) were compatible with EUS examination, to investigate extraesophageal causes of dysphagia in EUS examination, and to compare the esophageal wall thickness of achalasia patients with the healthy group.

## Materials and Methods

The study was conducted in accordance with the principles described in the Declaration of Helsinki and defined in Good Clinical Practice. The study was approved by the Local Ethics Committee of University of Health Sciences Bağcılar Training and Research Hospital (Decision no: 2020.10.1.05.151 Date: October 9, 2020). Patients were informed about the study in detail and their written and verbal consents were obtained. The study was conducted retrospectively in patients with dysphagia who presented to the gastroenterology outpatient clinic of University of Health Sciences Bagcilar Training and Research Hospital. The data used in the study were obtained from the information recorded in the hospital system during outpatient clinic visits. The study group consisted of 77 patients with dysphagia symptoms between the ages of 18 and 80. In both the patient and control groups, patients who were pregnant or lactating; who were allergic to contrast media; who had severe hepatic, renal, or cardiac insufficiency; who had previous upper gastrointestinal (GI) tract surgery; or who underwent endoscopic procedures such as pneumatic dilatation or botox injection were excluded. All patients included in the study were examined for motility disorders by HRM. Esophageal gastric duodenal graphy, upper GI endoscopy reports, HRM reports, and EUS reports of patients with dysphagia were analyzed. Among the 77 patients included, all patients were examined and evaluated for esophageal motor dysfunction. In the study, 69 patients had esophageal dysphagia. Eight patients described oropharyngeal dysphagia. Endosonography results were obtained in 27 of the total 29 achalasia patients included in the study.

High-resolution manometry examination was performed with a 22-channel aqueous system manometry catheter. The procedure started by asking the patients to fast for at least 6 hours and sending the catheter into the esophagus through the nose. The location of the upper and lower esophageal sphincter was determined, and the correct placement of the catheter was ensured. After measuring the resting pressures of the sphincters, 5 ml 10 times normal swallowing was done, then consecutive swallowing with 2 ml at 2 second intervals and rapid swallowing with 200 ml was done. Esophagogastric junction (EGJ) relaxation, contractile function, and pressurization properties of the esophagus were evaluated for each swallow by HRM. Endosonographic ultrasonography measurements were obtained from EGJ. Proximal esophageal dilatation was not present in the patients because they were early-stage patients.

The IRP cut-off value was selected as 20 mmHg Motility disorders of the patients were recorded according to Chicago classification version 4. According to the HRM examination results in the study, 28 of 29 patients diagnosed with achalasia were diagnosed as Type 2 achalasia according to Chicago classification, and 1 patient was diagnosed as Type 3 achalasia. Endosonographic ultrasonography procedure: It was performed with a Fujinon brand device using a radial probe. Esophageal wall thickness, total, muscularis propia, and muscularis mucosa thickness were measured using EUS. Mediastinal pathologies that could cause pressure on the esophagus were evaluated with EUS. Patients were evaluated with standard radial EUS in terms of extraesophageal findings and malignancy causing esophageal outlet obstruction, vascular compression, and lymphadenopathies.

### Statistical Analysis

Statistical analysis of the data was performed on a computer using IBM SPSS Windows version 21.0 (IBM SPSS Corp.; Armonk, NY, USA). In addition to descriptive statistical methods, whether the data set showed a normal distribution was determined by kurtosis and skewness measures, Kolmogorov–Smirnov, and Shapiro–Wilks tests. Chi-square was used for comparison between groups. The *t-*test was used to evaluate 2 groups with normal distribution, and the Mann–Whitney *U* test was used to compare 2 groups that did not. Pearson and Spearman’s correlation analysis tests were applied to determine the relationship. Analysis results were evaluated at the lowest significance level of *P* < .05 and the highest level of *P* < .01.

## Results

**Figure 1.**
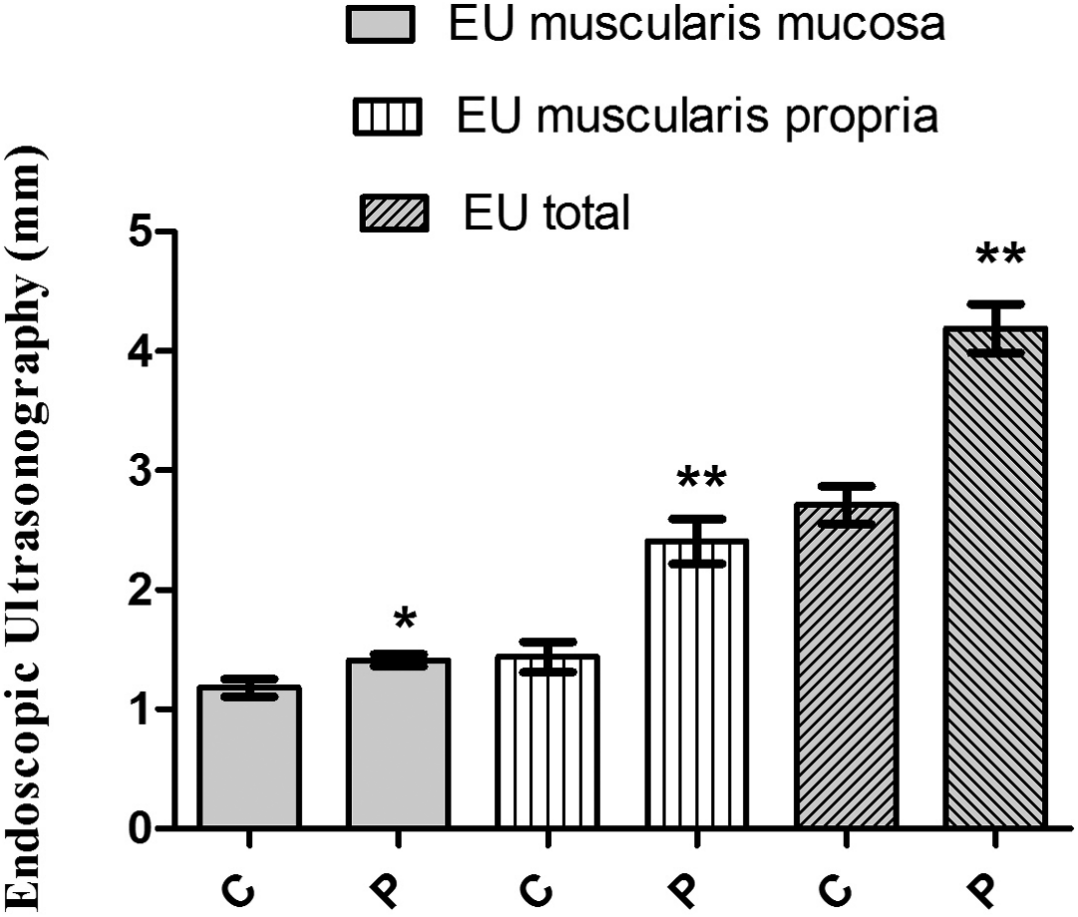
Endosonographic ultrasonography thickness findings of control and achalasia patient groups. *P < .05, **.*P* < .01 C, control; P, patient.

The mean age of the 77 patients with dysphagia in this study was 50 years, 41 were male (53%) and 36 were female. The characteristics of the patients are shown in Table 1. When the characteristics of 27 achalasia patients and 16 healthy control groups detected by HRM were analyzed, it was seen that there was no difference between the control and patient groups in terms of gender, age, and body mass index (BMI) (*P* > .05) (Table 2). IRP and endoscopic ultrasonography (EU) muscularis mucosa, EU muscularis propria, and EU total values of achalasia patients were higher than those of the healthy control group (*P* < .05, *P* < .01) (Table 2) (Figure 1).

**Figure 2.**
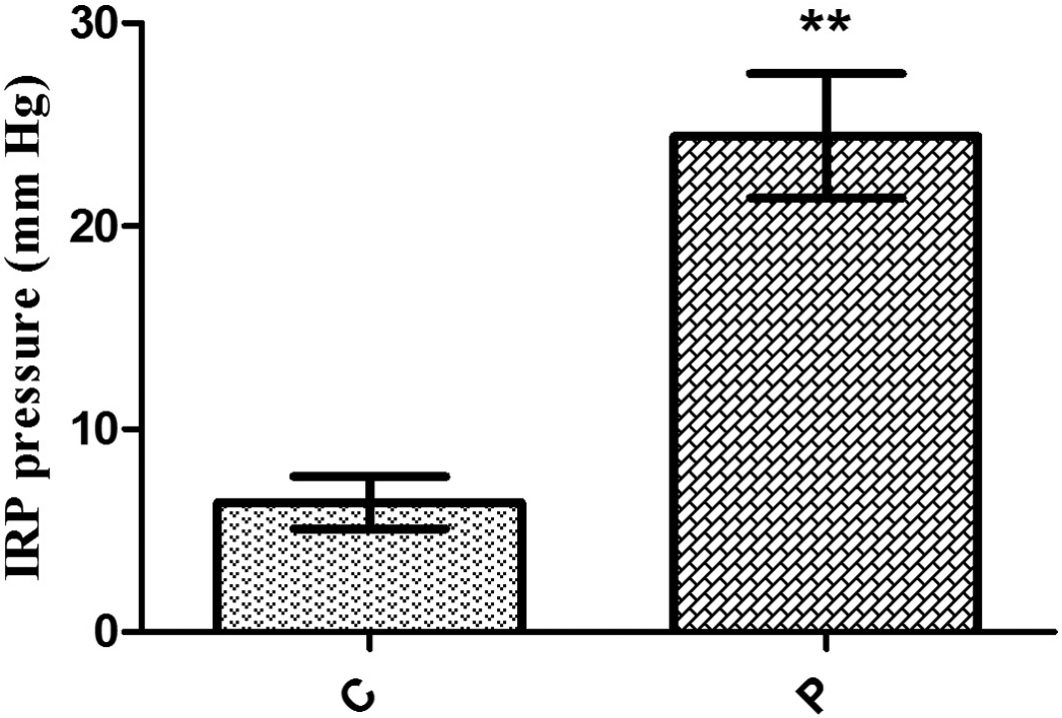
IRP pressure findings of the control and achalasia patient groups. **P < .01. C, control; P, patient.

It was observed that 59.3% of achalasia patients had IRP values above 20 mmHg. The mean disease duration was 5.82 ± 7.48 years, and the mean Eckardt scoring was 6.18 ± 2.2 (Table 3) (Figure 2). No statistically significant difference was found between Eckardt scoring, IRP, and EUS values between male and female achalasia patients (*P* > .05). (Table 4).

**Figure 3.**
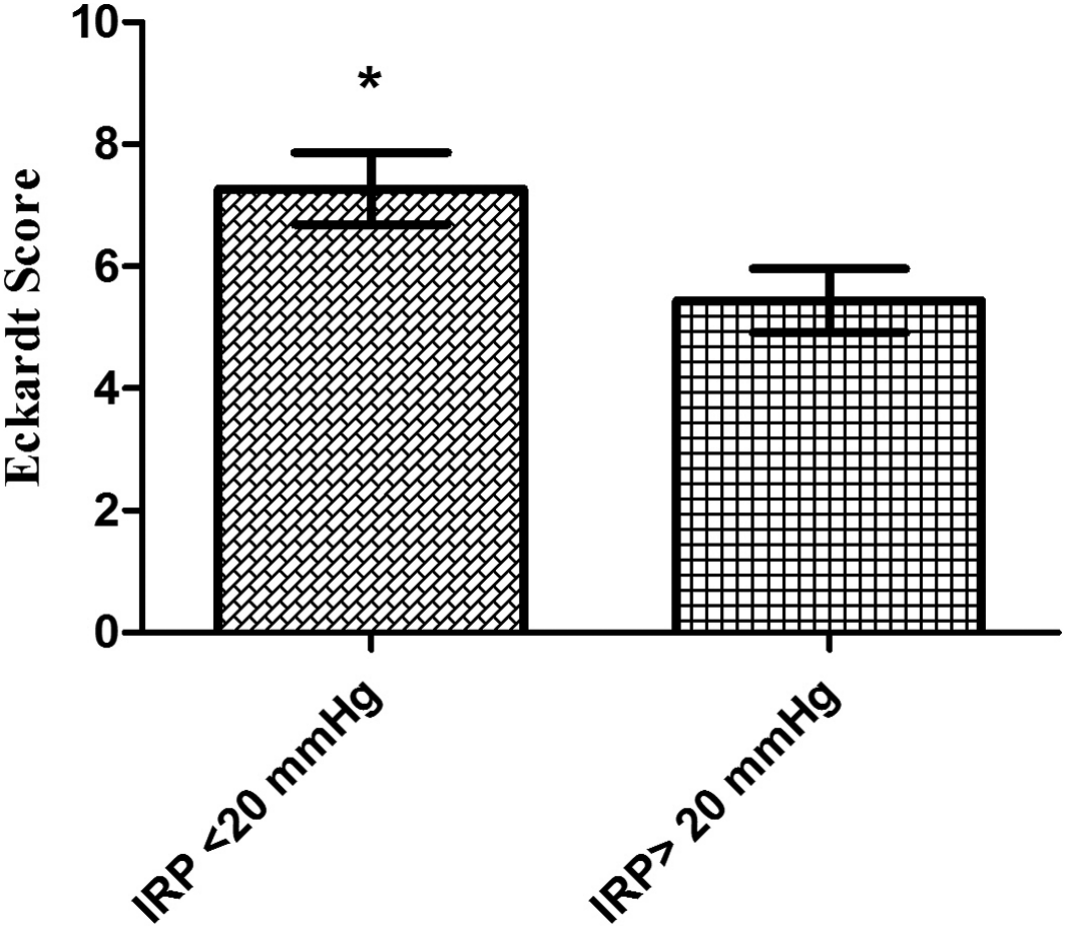
Distribution of Eckardt scoring values according to IRP pressure findings. *P < .05

In the control group, there was a positive correlation between age and EU muscularis mucosa values; a positive correlation was found between BMI and EU muscularis mucosa and muscularis propria (*P* < .05). No significant relationship was found between IRP and EUS values (*P* > .05). In the achalasia patient group, no significant relationship was found between age, BMI, disease duration, Eckardt scoring, IRP pressure values, and EU values (*P* > .05) (Table 5). Eckardt scoring and IRP pressure values of those with IRP pressure above 20 mmHg in the achalasia patient group were significantly higher than those with IRP pressure below 20 mmHg (*P* < .05, *P* < .01) (Table 6) (Figure 3). There was no difference in age, BMI, gender, disease duration, and EUS values between those with IRP pressure below 20 mmHg and those with IRP pressure above 20 mmHg in the achalasia patient group (*P* > .05) (Table 6).

## Discussion

Achalasia is an important health problem affecting the quality of life among the major esophageal motility disorders. Achalasia is considered a rare disease with an annual incidence of approximately 1.6 patients per 100 000 people and a prevalence of 10 patients per 100 000 people.^8^ Since it is a rare disease, patients are often misdiagnosed as having gastroesophageal reflux disease, other esophageal motor disorders, and psychiatric disorders.^9^ The diagnosis of achalasia is mainly made by HRM. Some patients diagnosed with achalasia on HRM may have pseudoachalasia and may have an underlying serious problem such as malignancy. Endosonography is a reliable diagnostic tool in the differential diagnosis of cardia tumor and achalasia.^10^ Patients with esophageal motility disorders were found to have increased esophageal muscle thickness on EUS compared with healthy subjects.^6^^,^^7^ In EUS examination of achalasia patients, it was found using manometry that esophageal circular, longitudinal, and total muscle layer thicknesses were significantly higher compared to normal volunteers.^11^ In this study, the aim was to compare the clinical usability of EUS in patients with dysphagia and the esophageal wall thickness of achalasia patients in the healthy group. Krishnan et al^12^ detected in the EUS examination of 24 patients diagnosed with achalasia on HRM, submucosal carcinoma in 1 patient and submucosal leiomyoma in 1 patient. Barthet et al^13^ detected pseudoachalasia in 2 cases in an EUS study with 35 achalasia patients. Devière et al^14^ found that 2 patients diagnosed with achalasia had pseudoachalasia due to tumor infiltration. In this study, although the HRM evaluation of 1 patient was compatible with the diagnosis of achalasia, a cardia tumor was detected in the EUS examination, and an esophageal tumor was detected in the EUS examination of 1 patient, whose motility disorder was not detected in the HRM examination. Mayberry et al^15^ determined that achalasia in 74 male and 78 female patients, and achalasia was similar in both genders. Sadowski et al^16^ found that 59.6% of 463 achalasia cases were male and 40.4% were female. In this study, it was determined that 55.6% of the patients complaining of dysphagia were male and 44.4% were female, and the diagnosis of achalasia was more common in men. Barthet et al^13^ showed the mean duration of dysphagia complaint in achalasia patients as 4.89 years. In this study, the mean duration of the disease was found to be 5.82 ± 7.48 years. Barthet et al^13^ measured esophageal total wall thickness at the cardia level of the control group without esophageal disease as 2.78 mm, while it was found to be 3.15 mm in achalasia patients. The thickness of the muscularis propia at the cardia level of the control group was 0.85 mm, while the thickness of the muscularis propia at the cardia level of the patient group was 1.01 mm.^13^ Van Dam et al^17^ found that 5 of 17 achalasia patients had sigmoid esophagus structure and 12 patients had straight esophagus structure. They found that the esophageal wall thickness of patients diagnosed with achalasia on HRM increased compared to healthy subjects. In this study, the esophageal total wall thickness measured at the level of the cardioesophageal junction in the EUS examinations of patients diagnosed with achalasia by HRM examination was 4.19 ± 1.05 mm, and the esophageal wall thickness measured at the level of the cardioesophageal junction in the EUS examination of the control group consisting of 16 patients who were not diagnosed with esophageal motility disorder in HRM examination was 2.71 ± 0.63.

Kim et al^18^ 24 of 89 achalasia patients had normal IRP values (IRP < 20).^18^ When the group with normal IRP value and the group with high IRP value before Peroral endoscopic myotomy treatment were compared in terms of gender variables, the number of males and females in the group with normal IRP value was 11 and 13, respectively; among achalasia patients with high IRP value, the number of males and females was 34 and 31, respectively, and there was no significant correlation between IRP value and gender variables. In this study, in the group with IRP pressure below 20 mmHg, the number of female patients was 4 (33.3%), the number of male patients was 7 (46.7%), and in the group with IRP pressure above 20 mmHg, the number of female genders was 8 (66.7%), the number of male genders was determined as 8 (53.3%). The study conducted by Kim et al^18^ found that the average age of achalasia patients with normal IRP values (51.12 ± 15.51) was higher than the average age of achalasia patients with high IRP values (39.80 ± 13.82).^18^ In this study, the average age of the group with normal IRP pressure was calculated as 44.63 ± 15.29, and the average age of the group with high IRP pressure was calculated as 45.25 ± 10.16. Kim et al^18^ found that the BMI of achalasia patients with normal IRP values was 22.97 ± 3.42, that of achalasia patients with high IRP values was 22.28 ± 3.36, and that there was no significant correlation between the IRP value and the BMI variable. In this study, BMI was 25.22 ± 3.89 in the group with normal IRP pressure, BMI was 26.66 ± 3.26 in the group with high IRP pressure, and the disease duration of the group with normal IRP value was 6.31 ± 6.92 years. The disease duration of achalasia patients with high IRP values was calculated as 3.94 ± 5.65 years and they found that the symptom duration of the group with normal IRP values was longer.

In this study, the disease duration of the group with normal IRP pressure was 6.18 ± 9.27, and the disease duration of the group with high IRP pressure was 5.57 ± 6.29. In Kim et al’s^18^ study, they calculated the Eckardt score of patients with normal IRP values to be 6.47 ± 2.87, and the Eckardt score of the achalasia group with high IRP values to be 6.48 ± 2.28. They found that there was no significant correlation between the IRP value and the Eckardt score.^18^ In this study, it was determined that the Eckardt score of the group with normal IRP pressure was 7.27 ± 1.95, and the Eckardt score of the group with high IRP pressure was 5.43 ± 2.09.

There was no significant correlation between the 2 groups, with EUS showing that patients with normal IRP values had a wall thickness of 2.96 ± 1.24 mm at the esophagogastric junction, and patients with achalasia with high IRP values having a wall thickness of 2.64 ± 1.10 mm at the esophagogastric junction. The muscularis mucosa thickness of achalasia patients with normal IRP values was measured as 1.72 ± 0.95 mm, the muscularis mucosa thickness of achalasia patients with high IRP values was measured as 1.49 ± 0.82, and there were differences in muscularis mucosa and esophagogastric junction wall thicknesses between both groups. They found that there was no significant difference.^18^ In this study, the esophageal muscularis mucosa thickness was measured as 1.46 ± 0.21 mm, the muscularis propia thickness was 2.51 ± 0.98 mm, and the esophageal total wall thickness was 4.11 ± 1.26 mm in the EUS examination of those with normal IRP pressure. In the EUS examination of those with high IRP pressure, esophageal muscularis mucosa thickness was measured as 1.44 ± 0.29 mm, esophageal muscularis propia thickness was 2.5 ± 0.8 mm, and esophageal total wall thickness was measured as 4.24 ± 0.92 mm. Additionally, no difference was found between those with normal and high IRP pressure in terms of age, BMI, gender, disease duration, and EUS values (*P* > .05). Kim et al^18^ showed that no significant relationship between the Eckardt score in the group with high and normal IRP pressure values. It has been found that those with normal IRP pressure are older and have a longer disease duration. In this study, although there was no significant relationship between the normal IRP pressure group and the high IRP pressure group, it was found that the disease duration was longer in the group with normal IRP pressure. It was found only 1 study in the literature comparing achalasia patients with normal IRP pressure and high IRP pressure. Comparative studies on larger patient samples may show the effect of IRP pressure value on Eckardt’s score. In this study, cardia tumor was detected in 1 patient diagnosed with achalasia in HRM performed due to dysphagia and chest pain, and an esophageal tumor was detected in the EUS examination of a patient with normal HRM and dysphagia. In addition, esophageal muscularis mucosa, muscularis propria, and total wall thicknesses were found to be significantly increased in the EUS examination of patients with achalasia on HRM compared to the healthy group. In the literature, there are few studies comparing EUS esophageal wall thicknesses of achalasia patients with high IRP pressure and normal groups. In this study, Eckardt’s score was significantly higher in the group with normal IRP pressure compared to the group with high IRP pressure. In addition, although not statistically significant, the disease duration was longer in the group with normal IRP. Although no significant correlation was found between EUS wall thickness increases in both groups, prospective studies in more patients are needed.

Although esophageal gastroduodenal radiography, manometry, and upper gastrointestinal endoscopy are the first preferred diagnostic methods in the diagnosis of dysphagia, it is known that these diagnostic methods are insufficient in terms of identifying pathologies that compress the esophagus from the outside. Endosonography has been found to differentiate achalasia and pseudoacalasia, to help change the current treatment and follow-up especially when malignancy is detected in the group of patients diagnosed with pseudoacalasia, and to be an imaging technique that has an important role in the evaluation of pathologies compressing the esophagus from the outside.

Endosonographic ultrasonography technology has been rapidly evolving over the last 10 years, allowing for improved diagnostic and therapeutic interventions. One study reported that EUS is the most accurate method for staging esophageal cancer. It is proposed as a minimally invasive procedure that uses high-frequency sound waves to visualize the layers of the esophageal wall and surrounding tissues, thus evaluating the primary tumor as well as locoregional adenopathy. With this study, it is suggested that EUS may help the diagnosis of early esophageal cancer by showing asymmetric wall thickness increase in the esophageal wall.^19^

In this study, malignancy was diagnosed with a rate of 2.59% among patients presenting with dysphagia, and it is thought that this rate would be higher in prospective, multicenter studies with large patient participation. In this study, when the healthy group was compared with the group diagnosed with achalasia, there was a significant increase in esophageal wall thickness measured at the cardioesophageal junction in patients diagnosed with achalasia, thus correlating with the results obtained in previous studies. It may be useful to perform manometric evaluation in terms of motility disorder in patients with increased esophageal wall thickness on EUS during the investigation of upper GI pathologies.

## Figures and Tables

**Table 1.** Demographics and Clinical Characteristics of the Study Sample (N = 77)

Gender	Male: 41
	Female: 36
Symptom	Dysphagia: 76
	Chest pain: 21
Causes of dysphagia according to manometry results	Achalasia: 29 Distal esophageal spasm: 4 Jackhammer esophagus: 2 EGJ outlet obstruction: 2 Ineffective motility disorder: 1
Causes of dysphagia other than manometry results	Drug-related dysphagia (nsaii, aspirin): 11 Functional dysphagia: 7 Gastroesophageal reflux disease: 6 Malignancy: 3 Osteophyte compression: 3 Cervical hernia surgery. secondary: 2 Thyroid gland anomaly: 2 Hiatal insufficiency: 2 Diverticula: 1 Schatzki ring: 1 Aortic compression: 1
Causes of dysphagia according to EGD results	Achalasia: 23 Osteophyte compression: 4 Aortic compression: 3 Diverticula: 3 Hiatal hernia: 2 Mass/lesion compression: 2 Cervical vertebra blockage: 1 Cricopharyngeal muscle hypertrophy: 1
Causes of dysphagia according to endoscopy results	Hiatal insufficiency: 9 Diverticula: 1 Schatzki ring: 1 Mallory Weiss scar in esophagus: 1 Malignancy: 1

EGD, esophageal gastric duodenal graphy; GI, gastrointestinal.

**Table 2.** Characteristics of the Control and Achalasia Patient Groups

Variable	Group	n	%	Test Values
Gender	Control male	9	56.2	*χ*2 = 0.56 *P *= .45
	Control female	7	43.8	
	Patient male	15	55.6	
	Patient female	12	44.4	
Variable	Group	Mean ± SD	Minimum-Maximum	Test Values
Age	Control (n = 16)	48 ± 15.87	18-72	t = 0.69; *P* = .49
	Patient (n = 27)	45 ± 12.23	21-67	
BMI	Control (n = 16)	26.66 ± 5.88	16.61-35.25	t = 0.39; *P* = .69
	Patient (n = 27)	26.07 ± 3.54	17.28-32.03	
IRP (mmHg)	Control (n = 16)	6.38 ± 5.16	0-18	Z = 4.85; *P* = .000**
	Patient (n = 27)	24.44 ± 15.93	10-88	
EU muscularis mucosa	Control (n = 16)	1.18 ± 0.29	0.37-1.6	t = 2.65; *P* = .011*
	Patient (n = 27)	1.41 ± 0.26	1-2	
EU muscularis propria	Control (n = 16)	1.48 ± 0.42	1-2	t = 4.42; *P* = .000**
	Patient (n = 27)	2.5 ± 0.86	1-4	
EU total	Control (n = 16)	2.71 ± 0.63	1.84-4.4	t = 5.07; *P* = .000**
	Patient (n = 27)	4.19 ± 1.05	2.4-6.24	

BMI, body mass index; EU, endoscopic ultrasonography; IRP, integrated relaxation pressure; Z, Mann–Whitney *U* test; *t*, *t* test value; χ2, ki-kare test value.*P < .05, **P < .01.

**Table 3.** Other Characteristics of the Achalasia Patients

Variable	n	%
IRP below 20 mmHg	11	40.7
IRP above 20 mmHg	16	59.3
	Mean ± SD	Minimum-Maximum
Disease duration	5.82 ± 7.48	5 months-30 years
Eckardt scoring	6.18 ± 2.2	2-11

**Table 4.** Comparison of Eckardt Scoring, IRP, EU Values of Achalasia Patients According to Gender

Variable	Female	Male	Test Values
Eckardt scoring	6.83 ± 2.40	5.66 ± 1.95	*t* = 1.39 *P* = .17
IRP (mmHg)	21.41 ± 9.14	26.86 ± 19.78	Z = 0.61 *P* = .54
EU muscularis mucosa	1.33 ± 0.32	1.46 ± 0.2	*t* = 1.29 *P* = .20
EU muscularis propria	2.67 ± 0.91	2.28 ± 0.77	*t* = 1.16 *P* = .25
EU total	4.31 ± 1.05	4.03 ± 1.08	*t* = 0.68 *P* = .49

EU, endosonographic ultrasonography; IRP, integrated relaxation pressure; Z, Mann–Whitney *U* test; *t*, *t* test value.

**Table 5.** Examination of the Relationship Between Variables and EU Values in the Control and Patient Groups

	EU Muscularis mucosa	EU Muscularis Propria	EU Total
Control group			
Age	*r* = 0.59* *P* = .015	*r* = 0.39 *P* = .13	*r* = 0.35 *P* = .17
BMI	*r* = 0.52* *P* = .03	*r* = 0.60* *P* = .013	*r* = 0.36 *P* = .16
IRP	*r* = 0.30 *P* = .25	*r* = 0.31 *P* = .24	*r* = 0.07 *P* = .78
Patient group			
Age	*r* = 0.82 *P* = .68	*r* = 0.13 *P* = .5	*r* = 0.15 *P* = .42
BMI	*r* = 0.23 *P* = .24	*r* = 0.27 *P* = .16	*r* = 0.22 *P* = .25
Duration of disease	*r* = 0.06 *P* = .73	*r* = 0.09 *P* = .63	*r* = 0.24 *P* = .21
Eckardt scoring	*r* = 0.22 *P* = .27	*r* = 0.15 *P* = .43	*r* = 0.02 *P* = .89
IRP	*r* = 0.20 *P* = .31	*r* = 0.21 *P* = .28	*r* = 0.17 *P* = .39

Spearman’s vs Pearson correlation tests, r = correlation value.

BMI, body mass index; EU, endosonographic ultrasonography; IRP, integrated relaxation pressure.

*P < .05.

**Table 6.** Comparison of Age, BMI, Gender, Disease Duration, Eckardt Scoring, IRP, and EU Values of Achalasia Patients According to the IRP

Variable	IRP < 20 mmHg Mean ± SD	IRP > 20 mmHg Mean ± SD	Test Values
Age	44.63 ± 15.29	45.25 ± 10.16	*t* = 0.12 *P* = .9
BMI	25.22 ± 3.89	26.66 ± 3.26	*t* = 1.04 *P* = .3
Gender Male Female	n = 7 (46.7%) n = 4 (33.3%)	n = 8 (53.3%) n = 8 (66.7%)	*X*^2^ = 0.49 *P* = .48
IRP (mmHg)	13.72 ± 9.14	31.81 ± 17.12	*Z* = 4.34 *P* = .000**
Duration of disease	6.18 ± 9.27	5.57 ± 6.29	*Z* = 0.12 *P* = .90
Eckardt scoring	7.27 ± 1.95	5.43 ± 2.09	*t* = 2.29 *P* = .03*
EU muscularis mucosa	1.46 ± 0.21	1.44 ± 0.29	*t* = 0.84 *P* = .4
EU muscularis propria	2.51 ± 0.98	2.5 ± 0.8	*t* = 0.03 *P* = .97
EU total	4.11 ± 1.26	4.24 ± 0.92	*t* = 0.29 *P* = .77

BMI, body mass index; EU, endoscopic ultrasonography; t, t test; Z, Mann–Whitney *U* test; χ2, chi-square text.

*P < .05, **P < .01.

## Data Availability

All data generated or analyzed during this study are included in this article. Further enquiries can be directed to the corresponding author.
